# Short- and Midterm Results between Laparoscopic Roux-en-Y Gastric Bypass and Laparoscopic Sleeve Gastrectomy for the Treatment of Morbid Obesity

**DOI:** 10.1155/2013/934653

**Published:** 2013-09-02

**Authors:** Bandar Albeladi, Céline Bourbao-Tournois, Noel Huten

**Affiliations:** Department of Digestive and Bariatric Surgery, TOURS University Hospital (Hôpital Trousseau), Avenue de la République, Chambray lès Tours, 37170 Tours, France

## Abstract

*Background*. Laparoscopic Roux-en-Y gastric bypass (LRYGB) is one of the most widely used bariatric procedures today, and laparoscopic sleeve gastrectomy (LSG) as a single-stage procedure for the treatment of morbid obesity is becoming increasingly popular in Europe. The aim of this study was to compare short- and midterm results between LRYGB and LSG. *Methods*. An observational retrospective study from a database of patients undergoing LRYGB and LSG between January 2008 and June 2011. Seventy patients (mean age 39 years) were included. Patients were followed at 6, 12, and 18 months. Operative time, length of stay, weight loss, comorbidity improvement or resolution, postoperative complications, reinterventions and mortality were evaluated. *Results*. Thirty-six LRYGB and 34 LSG were included. Mean operative time of LSG was 106 min while LRYGB was 196 min (*P* < 0.001). Differences in length of stay, early and late complications, and improvement or resolution in comorbidities were not significant (*P* > 0.05). Eighteen months after surgery, average excess weight loss was 77.6% in LRYGB and 57.1% in LSG (*P* = 0.003). There was no surgery-related mortality. *Conclusions*. Both LRYGB and LSG are safe procedures that provide good results in weight loss and resolution of comorbidities at 18 months.

## 1. Introduction

The obesity epidemic continues to increase worldwide and is associated with many comorbidities resulting in increased mortality rates of obese people [1, 2].

These comorbidities not only lead to a reduction in life expectancy, but also in quality of life [3]. And surgery remains the only proven treatment modality [4, 5].

Presently advocated Laparoscopic Roux-en-Y gastric bypass (LRYGB) is the most frequently performed bariatric procedure providing significant and sustained weight loss at long-term followup [6, 7].

The laparoscopic sleeve gastrectomy (LSG), initially used in patients with high surgical risk as the first stage of a more complex procedure (duodenal switch or gastric bypass), has gained popularity in recent years due to reported good short-term results and its relatively lower technical complexity [5–10].

However, the long-term efficacy is under investigation and there are very few studies that compare it with other bariatric techniques, including LRYGB.

The purpose of this study was to retrospectively compare the early results of LRYGB and LSG for a period of 18 months and follow them up over a period of 5 years to see if LSG can replace LRYGB as the gold standard bariatric procedure in France.

## 2. Material and Methods

The study was done at the university hospital in Tours (Department of Digestive and Bariatric Surgery). It is a retrospective observational study.

The study group included patients who were operated on between January 2008 and June 2011. The inclusion criteria for the study were (1) BMI > 40 or BMI > 35 with a significant comorbidity associated with morbid obesity (type 2 diabetes, hypertension, obstructive sleep apnea, dyslipidemia, and arthrosis), (2) age between 18–60 years old, and (3) previous successfully instituted and supervized but failed adequate diet and exercise program, in accordance with the French national guidelines (HAS).

The exclusion criteria were significant psychiatric disorder, severe eating disorder (binge eating), active alcohol or substance abuse, active gastric ulcer disease, difficult GERD with a large hiatal hernia, and previous bariatric surgery (except gastric banding).

### 2.1. Preoperative Evaluation

All the patients underwent evaluation by a bariatric multidisciplinary team (endocrinologist, dietitians, psychiatrist, anaesthesiologist, and surgeons) and in accordance with the French national guidelines. Candidates for surgery were informed about the procedure and they completed an extensive preoperative workup indicated by the multidisciplinary group.

Upper gastrointestinal endoscopy and abdominal ultrasound examination were performed on all patients. Possible *Helicobacter pylori* infection and associated gastric ulcer disease were treated and controlled after that before surgery.

### 2.2. Surgical Technique

#### 2.2.1. Laparoscopic Sleeve Gastrectomy

The four-port technique was used. The gastrosplenic omentum was divided from the greater curvature close to the stomach wall using Ultracision or a Ligasure device. The left crus of the diaphragm was completely dissected and clearly visualized and the angle of His delineated. Posterior adhesions to the pancreas were dissected.

After leaving 6 cm of antrum from the pylorus, the sleeve of the stomach was created over a 36-Fr (12 mm) gastric calibration tube. Firing of the linear stapler was done using a gold loads (3.8 mm staples) without any buttress material to reinforce it. The methylene blue test was performed to check for a leak. A specimen of the stomach was removed. Cholecystectomy was performed for symptomatic gallstones.

#### 2.2.2. Laparoscopic Roux-en-Y Gastric Bypass

An antecolic and antegastric Roux-en-Y gastric bypass was performed with an alimentary limb of 150 cm. Biliopancreatic limb was 75 cm in all cases. A side-to-side jejunojejunostomy was done using linear stapler with white loads (2.5 mm staples). An omental split was done. A 20–30 cm^3^ vertical gastric pouch was created over a 36-Fr (12 mm) gastric calibration tube, without leaving any posterior pouch. End-to-side gastrojejunostomy was done using absorbable surgical suture. At the end of the procedure, the methylene blue test was injected to identify possible leaks. Mesenteric and Petersen defect was sutured in all cases with nonabsorbable surgical suture. A closed suction drain was placed in the proximity of the gastrojejunostomy. Cholecystectomy was performed for all gallstones.

#### 2.2.3. Demographic Data

All patients were informed in detail about the risk and benefits of each technique. Indication for LRYGB or LSG was based on clinical criteria and the consensus of the bariatric surgery unit. The patients were matched for age, gender, and body mass index (BMI). The demographic data of the two groups are listed in Table 1.

A statistically significant difference in BMI was observed between both groups related to the inclusion of superobese patients in the LSG group.

Both groups were evaluated in terms of weight loss, resolution of comorbidities, and complications at 6, 12, and 18 months. Mean percent EWL and mean BMI were calculated. Complications were defined as early (<30 days) and late (>30 days).

Patient compliance with their scheduled follow-up visit for both procedures was 100% at 6 months, 98.5% at 12 months, and 90% at 18 months.

#### 2.2.4. Comorbidities

In the preoperative assessment, type 2 diabetics were 19.4% (*n* = 9) in the LRYGB group and 2.9% (*n* = 1) in the LSG group. The date of the first diagnosis was not known in the majority of diabetics patients. 41.7% (*n* = 15) of patients in the LRYGB group had hypertension and 38.2% (*n* = 13) of those in the LSG group were suffering from hypertension. Hiatus hernia was 22.2% (*n* = 8) in the LRYGB group and 0% in the LSG group because, in our hospital, we prefer not to perform LSG for patients who had symptomatic hiatus hernia.

In the LRYGB group, 13.9% (*n* = 5) of the patients were suffering from joint pains compared to 11.8% (*n* = 4) in the LSG group. Sleep apnea were 36.1% (*n* = 13) in the LRYGB group and 26.5% (*n* = 9) in the LSG group (Table 2).

The two groups were found similar in past surgical history and use of medications. 

## 3. Results

All procedures were done laparoscopically but three (4%), where conversions to open surgery were needed, two in the LRYGB who had gastric banding before, and one in the LSG group.

The median operating time for the LRYGB group of 196 min was significantly longer than that for the LSG group of 106 min (*P* < 0.001).

The median length of hospitalization was 7 days in the LRYGB group and 6 days in the LSG group. The satisfaction of the patients assessed by the medical team was 97% in the LRYGB group and 91% in the LSG group Table 3.

### 3.1. Clinical Outcome: Weight Loss

There was a significant difference in mean percentage of excess weight loss (EWL) between LRYGB and LSG (Table 4).

**Figure 1:**
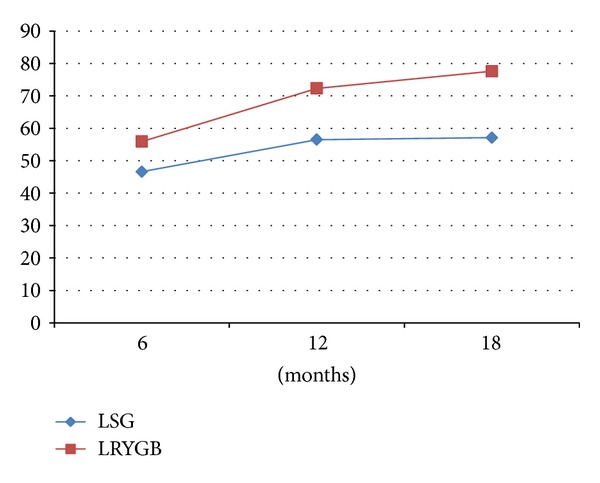
Evolution of percentage of excess weight loss (%EWL) after bariatric surgery: laparoscopic Roux-en-Y gastric bypass (LRYGB) versus laparoscopic sleeve gastrectomy (LSG).

The mean Percent of EWL at the end of 6 months was 46.6% in the LGS group and 55.9% after LRYGB. At the end of 12 months, the mean EWL was 56.5% in the LSG group and 72.3% in the LRYGB group. At 18 months, it was 57.1% in the LSG group and 77.6% in the LRYGB group (Figure 1).

**Figure 2:**
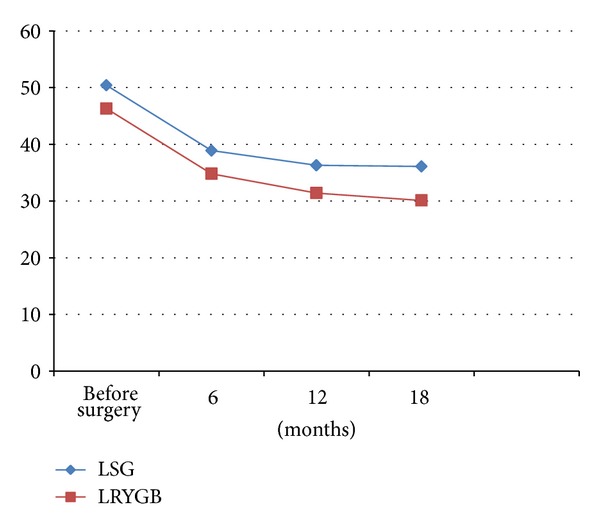
Evolution of body mass index (BMI) after bariatric surgery: laparoscopic Roux-en-Y gastric bypass (LRYGB) versus laparoscopic sleeve gastrectomy (LSG).

Similarly, a significant difference in the BMI was observed between LRYGB and LSG. At 6 months, the mean BMI was 38.9 kg/m^2^ in the LSG group and 34.8 kg/m^2^ in the LRYGB group. At 12 months, mean BMI was 36.3 kg/m^2^ in the LSG group and 31.4 kg/m^2^ in the LRYGB group. At 18 months, mean BMI was 36.1 kg/m^2^ in the LSG group and 30.1 kg/m^2^ in the LRYGB group (Figure 2).

### 3.2. Clinical Outcome. Resolution or Improvement of Comorbidities

Type 2 diabetes was resolved in 100% of patients in the LSG group. In the LRYGB group, type 2 diabetes was reported as resolved in 85.7%. In the remaining 14.3%, the dosage of medication was decreased. Resolution was considered as normal premeal and postmeal blood sugar levels without any medications.

**Figure 3:**
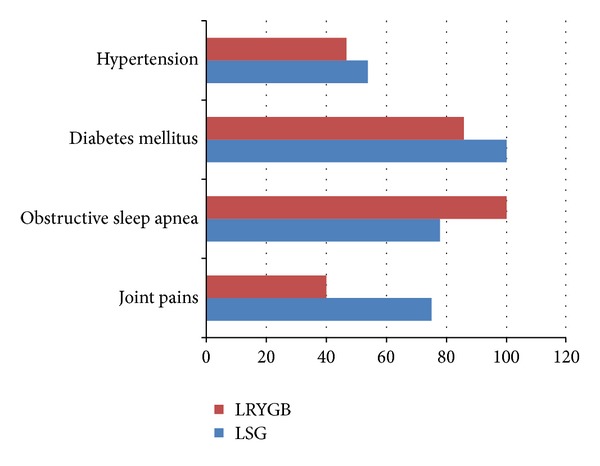
Resolution of Comorbidities after bariatric surgery: laparoscopic Roux-en-Y gastric bypass (LRYGB) versus laparoscopic sleeve gastrectomy (LSG).

Hypertension was resolved in 53.8% of patients who underwent LSG and in 46.7% of those who underwent LRYGB. Joint pains resolved in 75% of LSG group and 40% of the LRYGB group. Obstructive sleep apnea was resolved in 77.8% LSG group and in 100% of the LRYGB group. No significant differences were observed between the study groups in resolution of comorbidity (Figure 3).

### 3.3. Clinical Outcome. Complication

There was no mortality at the 12 months postoperatively. The overall 30-day morbidity (early complication) was 25% (*n* = 9) in the LRYGB group and 8.8% (*n* = 3) in the LSG group. One patient in the LSG group developed a staple line leak. It was treated by laparoscopic external drainage without stent. In the LRYGB, there was gastrojejunostomy leak in four patients; three were treated by laparoscopic external drainage and the fourth one by medical treatment. All the early and late postoperative complications are detailed in Table 5.

## 4. Discussion

LRYGB is a safe and effective bariatric procedure with excellent results reported over long-term followup. There is a significant weight loss and favorable effect on comorbidities. It is considered to be the procedure of choice the world over [11].

However, in recent years, LSG has been identified as an innovative approach to the surgical management of morbid obesity. It has attracted interest among surgeons as it is considered easier and faster to perform and less traumatic compared to LRYGB. Its advantages include preservation of endoscopic access to the upper gastrointestinal tract, the lack of an intestinal anastomosis thus excluding the risk of internal herniation, normal intestinal absorption, and prevention of the dumping syndrome due to pylorus preservation.

LSG was initially started as the first stage of a duodenal switch (DS) surgery. The rationale of a first-stage surgery in superobese patients was to achieve a substantial weight loss and amelioration of comorbidities, thus making the second-stage surgery a much safer surgery with a good chance to achieve the optimum weight loss. LRYGB and DS are the two surgeries that are commonly performed after a first-stage LSG. DS as a procedure is not very popular in France because it is difficult to follow up. LRYGB is the preferred procedure of choice for the second stage in our hospital.

Interestingly, in three studies that were intended to have a second-stage surgery, only 25% of patients finally had the second surgery. The second stage was not required in a good number of patients [12–14].

Some factors have been proposed to have influence in the percentage of EWL in LSG. Bougie sizes ranging from 32 to 60 F have been studied, but no direct correlation with percentage of EWL has been demonstrated [15]. The distance from the pylorus to the beginning of the gastric transection and the complete resection of the fundus responsible for the ghrelin secretion have been also proposed as factors influencing the results. However, there was no broad agreement on these technical aspects. In our study, the same maneuvers were used in all patients: after leaving 6 cm of antrum from the pylorus, the sleeve of the stomach was created over a 36-Fr gastric calibration tube. We take a special care in the dissection of the left crus, the identification of the fat pad, and the dissection of the posterior attachments from the stomach to the pancreas to facilitate the complete resection of the fundus and avoid any remnant that may cause failure as has been suggested by several authors [16, 17]. Perhaps this combination may account for the excellent results obtained with LSG.

The LSG is technically less complex than the LYRGB, which is reflected by a lower operative time in our series (*P* < 0.05). However, these characteristics are not translated into shorter postoperative hospital stay.

Although most of the available data suggest that morbidity related to LSG is lower than in LRYGB [10, 18–20]. And our results confirm that morbidity is lower in patients undergoing LSG, early and late complications in both groups showed no statistically significant difference (*P* > 0.05).

When only patients who achieved an EWL > 50% were considered, the results of this series show that both LRYGB and LSG were safe and effective bariatric procedures resulting in significant weight loss (LRYGB = 77% versus LSG = 57%), confirming data provided by other authors [18, 21–23].

Restriction of food intake and changes in appetite and satiety due to alterations in gut hormones are probably key mechanisms for weight loss after both procedures [24–26]. LSG and LRYGB are effective in terms of weight loss by simple restriction in combination with changes in gut hormones [24, 26]. Delivery to the jejunum of undigested chime of higher pH could enhance Peptide YY (PYY) and Glucagon -like peptide-1 (GLP-1) response to the meal, inducing satiety [24–26].

Decreased appetite seems to play a role in weight loss after LRYGB and LSG due to suppression of the orexigenic hormone ghrelin after elimination of gastric fundus [24–27].

In our study, the resolution of comorbidities is also favorable. That may become higher than 90% in diabetes mellitus or obstructive sleep apnea and more than 50% in hypertension. There are few data in the literature [19, 28, 29] comparing results between LRYGB and LSG, but the present data show similar resolution rate with both surgical techniques.

We suggested that the use of LSG as a definitive procedure for the surgical treatment of morbid obesity is a good option for the obese patient who does not have GERD or hiatus hernia. It is rapidly gaining popularity as a standalone procedure in France.

The limitations of our study are that it is a retrospective observational study. There is a selection bias as randomization was not possible. These are short- and midterm results in a small patient pool and the same surgical team treated all patients.

Secondly, our followup is limited to 18 months. Further randomized controlled studies are needed to elucidate long-term results, especially on the efficacy of the LSG as definitive bariatric procedure and to light up for mechanisms responsible for the success or failure in weight control and comorbidities resolution.

In conclusion, both LRYGB and LSG are safe procedures that provide good results in weight loss and resolution of comorbidities at 18 months. However, randomized studies with long-term followup with a larger patient pool are needed before we can recommend LSG as a standalone procedure.

## Figures and Tables

**Table 1:** Patients demographic data.

Characteristics	LRYGB	LSG	*P* value
Mean age (years)	39.7 ± 9.8 (21–64)	38.3 ± 11.3 (22–61)	*P* > 0.05
Mean BMI (kg/m^2^)	46.31 ± 5.95 (36.4–58.3)	50.39 ± 6.26 (40–63.6)	*P* = 0.006
Sex No. (%)	27 (75) F	28 (82) F	*P* > 0.05
	9 (25) M	6 (18) M	

LRYGB: laparoscopic Roux-Y gastric bypass, LSG: laparoscopic sleeve gastrectomy, BMI: body mass index.

**Table 2:** Patients comorbidities at baseline.

Comorbidity	LRYGB Number Of Pts (%)	LSG Number of Pts (%)	*P* value
Hypertension	15 (41.7)	13 (38.2)	*P* > 0.05
Diabetes Mellitus	7 (19.4)	1 (2.9)	*P* = 0.033
Joint pain	5 (13.9)	4 (11.8)	*P* > 0.05
Sleep apnea	13 (36.1)	9 (26.5)	*P* > 0.05

LRYGB: laparoscopic Roux-Y gastric bypass, LSG: laparoscopic sleeve gastrectomy.

**Table 3:** Median operating time, hospital stay, and satisfaction of the patients.

	LRYGB	LSG	*P* value
Median operating time	196 min (132–331)	106 min (52–224)	*P* < 0.001
Hospital stay	7 days (5–23)	6 days (4–59)	*P* > 0.05
Satisfaction	97%	91%	*P* > 0.05

LRYGB: laparoscopic Roux-Y gastric bypass, LSG: laparoscopic sleeve gastrectomy.

**Table 4:** Preoperative and postoperative comparison of weight loss and %EWL.

	LRYGB *N* = 36	LSG *N* = 34	*P* value
BMI (kg/m)			
Preoperative	46.3 ± 5.9	50.4 ± 6.26	*P* = 0.006
Postoperative			
6M	34.8 ± 5.2	38.9 ± 5.5	*P* = 0.002
12M	31.4 ± 5.6	36.3 ± 5.8	*P* < 0.001
18M	30.1 ± 6.1	36.1 ± 7.2	*P* = 0.002
% EWL			
6M	55.9 ± 13.1	46.6 ± 16.1	*P* = 0.01
12M	72.3 ± 19	56.5 ± 19.7	*P* = 0.001
18M	77.6 ± 20.5	57.1 ± 23.5	*P* = 0.003

Body mass index (BMI), excess weight loss (%EWL), laparoscopic Roux-en-Y gastric bypass (LRYGB), and laparos copic sleeve gastrectomy (LSG).

**Table 5:** Postoperative early and late complications.

	Complication	(NO)	Management
Early			
LSG	Intraperitoneal bleeding	1	relaparoscopy
	Infection intra-abdominal	1	relaparoscopy
	Leak	1	relaparoscopy

LRYGB	Intraperitoneal bleeding	1	relaparoscopy
	Anastomotic stenosis	1	Endoscopy
	Leak	4	3 relaparoscopy and 1 medical treatment
	Intra-abdominal infection	1	Medical treatment
	Deep vein thrombosis	1	Medical treatment
	Compartment syndrome of the right lower limb	1	Fasciotomy
			*P* > 0.05

Late			
LSG	Vitamin deficiency	7	

LRYGB	Internal hernia	1	relaparoscopy
	Gastrogastric fistula	1	Laparotomy
	Vitamin deficiency	11	
			*P* > 0.05

LRYGB: laparoscopic Roux-Y gastric bypass, LSG: laparoscopic sleeve gastrectomy.
